# Analyzing Subnational Immunization Coverage to Catch up and Reach the Unreached in Seven High-Priority Countries in the Eastern Mediterranean Region, 2019–2021

**DOI:** 10.3390/vaccines12030285

**Published:** 2024-03-08

**Authors:** Kamal Fahmy, Quamrul Hasan, Md Sharifuzzaman, Yvan Hutin

**Affiliations:** 1 Universal Health Coverage (UHC)/Department of Communicable Disease Prevention and Control (DCD), Immunization, Vaccine Preventable Diseases and Polio Transition (IVP), World Health Organization Regional Office for the Eastern Mediterranean, Cairo 34222, Egypt; hasanq@who.int (Q.H.); sharifuzzamanm@who.int (M.S.); 2 Universal Health Coverage (UHC)/Department of Communicable Disease Prevention and Control (DCD), World Health Organization Regional Office for the Eastern Mediterranean, Cairo 11371, Egypt; hutiny@who.int

**Keywords:** subnational immunization coverage, under-immunized children, immunization inequality, zero-dose children, COVID pandemic

## Abstract

Yearly national immunization coverage reporting does not measure performance at the subnational level throughout the year and conceals inequalities within countries. We analyzed subnational immunization coverage from seven high-priority countries in our region. We analyzed subnational, monthly immunization data from seven high-priority countries. Five were Gavi eligible (i.e., Afghanistan, Pakistan, Somalia, Syria, and Yemen); these are countries that according to their low income are eligible for support from the Global Alliance on Vaccine and Immunization, while Iraq and Jordan were included because of a recent decrease in immunization coverage and contribution to the regional number of under and unimmunized children. DTP3 coverage, which is considered as the main indicator for the routine immunization coverage as the essential component of the immunization program performance, varied monthly in 2019–2021 before reaching pre-pandemic coverage in the last two months of 2021. Somalia and Yemen had a net gain in DTP3 coverage at the end of 2021, as improvement in 2021 exceeded the regression in 2020. In Pakistan and Iraq, DTP3 improvement in 2021 equaled the 2020 regression. In Afghanistan, Syria and Jordan, the regression in DTP3 coverage continued in 2020 and 2021. The number of districts with at least 6000 zero-dose children improved moderately in Afghanistan and substantially in Somalia throughout the follow-up period. In Pakistan, the geographical distribution differed between 2020 and 2021.Of the three countries with the highest number of zero-dose children, DTP1 coverage reached 109% in Q4 of 2020 after a sharp drop to 69% in Q2 of 2020. However, in Pakistan, the number of zero-dose children decreased to 1/10 of its burden in Q4 of 2021. In Afghanistan, the number of zero-dose children more than a doubled. Among the even countries, adaptation of immunization service to the pandemic varied, depending on the agility of the health system and the performance of the components of the expanded program on immunization. We recommended monitoring administrative monthly immunization coverage data at the subnational level to detect low-performing districts, plan catchup, identify bottlenecks towards reaching unvaccinated children and customize strategies to improve the coverage in districts with zero-dose children throughout the year and monitor progress.

## 1. Introduction

In 2011, the World Health Organization (WHO) has set a global plan to improve immunization coverage and reduce vaccine-preventable diseases. This Global Vaccine Action Plan [GVAP] proposed to strengthen immunization systems worldwide and achieve equitable access to vaccines for all populations. The plan set dual targets of 90% national coverage and 80% coverage for all districts within countries by 2020, using the third dose of Diphtheria, Pertussis and Tetanus [DPT] coverage as a marker [1]. The plan also emphasized the integration of immunization goals with the Sustainable Development Goals [SDGs] to ensure that vaccines contribute to overall health and development outcomes. In 2020, immunization partners adapted the global strategy for immunization as per the Immunization Agenda [IA] 2030 that focuses on leaving no one behind. IA2030 envisions a world where everyone, everywhere, at every age, fully benefits from vaccines to improve health and well-being [2]. Key strategies to address some challenges in implementing the IA2030 are (1) increasing the financing of health systems, (2) addressing vaccine hesitancy, (3) building a strong partnership and making decisions based on data. Data at the subnational level drive the decision to address immunization service delivery gaps at the district level. All countries commit to report on their immunization coverage, among other indicators, at the end of every year. The joint reporting form (JRF) is the basis of the globally approved immunization coverage through the WHO–UNICEF Estimates of National Immunization Coverage based on administrative reported coverage and available surveys (WUNEIC) [3]. However, yearly reporting on immunization coverage lacks the ability to measure performance at the subnational level throughout the year. Further, national coverage data often conceal inequalities in coverage and access within the country [4]. During the COVID-19 pandemic, it could not be used to measure continuity of immunization service delivery and subsequent recovery. In contrast, analyzing immunization coverage at the district level allows for targeted interventions to improve immunization rates and prioritize intervention to reduce disparities [5,6]. Subnational immunization coverage analysis gained a global interest to identify low-performing districts. In some countries, data are available at the subdistrict level which allows for better consideration of any reduction in immunization coverage throughout the year.

The WHO Eastern Mediterranean Region (EMR) is one of the six World Health Organization regions with the lowest immunization coverage, with significant disparities between and within its 22 countries. Several countries of the region face numerous economic, social, and political challenges that affect the delivery of immunization services. Factors such as armed conflicts, population displacement, poverty, weak health systems, and cultural beliefs contribute to disparities in immunization coverage and hinder efforts to reach vulnerable populations. In 2012, the Eastern Mediterranean Regional Office (EMRO) developed as a regional framework aligned with the GVAP: the Eastern Mediterranean Vaccine Action Plan (EMVAP). EMVAP took into consideration regional specificities, specific needs of Member States and the challenges that these countries faced [7]. EMVAP had put a specific focus on all countries reaching the DTP3 coverage at 90% at the national level and at least 80% at the district (subnational) level. In 2023, the EMR was developing the regional framework in coherence with the IA 2030. Challenges in vaccination coverage in the EMR are considerable. Prior to the pandemic, in the Eastern Mediterranean region, the mean percentages of routine vaccination coverage increased from 2015 to 2019 for five (38.5%) vaccines and decreased for eight (61.5%) vaccines. Vaccination coverage increases from 2015 to 2019 ranged from 0.2% for the DTP1 vaccine and MCV2 to 11.9% for the first dose of inactivated poliovirus vaccine. Vaccination coverage reductions from 2015 to 2019 ranged from −0.2% for five vaccines (DTP3 vaccine, the first dose of hepatitis B vaccine, the third dose of hepatitis B vaccine, the third dose of poliovirus vaccine, and the first dose of rubella-containing vaccine) to −5% for the last dose of rotavirus vaccine. While in 2022, the coverage for DTP3 and MCV1 were 84% and *83%, respectively [8]. In 2019, in EMR, 1.8 million children did not get their first dose of DTP [zero-dose children]. EMRO identified seven high-priority countries in the region. Five of these are Gavi eligible countries (i.e., Afghanistan, Pakistan, Somalia, Syria, and Yemen). Iraq and Jordan are included as well in the review process because of the decrease in their immunization coverage and contribution to the regional under and unimmunized children. Further, three of the seven priority countries (Pakistan, Afghanistan, and Somalia) report two-thirds of zero-dose children in the region. In addition, overall, 2.9 million children in all EMR countries in 2022 did not receive their third dose of DTP3.

Analyzing immunization coverage at the subnational level allows better tracking of unimmunized children [9]. Regular monitoring of monthly immunization coverage at the subnational level allows countries to take timely decisions sooner rather than later. The EMRO started to support countries in analyzing their immunization coverage at the lowest possible subnational level. In 2018, the EMRO decided at the regional level to start collecting monthly immunization coverage data at the subnational level from high-priority countries. The objective was to obtain a greater understanding of disparities in the coverage and access within each country and to identify areas where immunization services were not reaching the population effectively. In the meantime, in 2020, the EPI in the region had to face the COVID-19 pandemic and organize the recovery of immunization services. Countries reported differences in immunization performance, as reflected by different antigens coverage, with a primarily focus on DTP3 and MCV2 coverage. The pandemic impacted one or more of the components of the expanded program on immunization (e.g., governance, work force, finance, vaccine and cold chain, immunization service delivery, surveillance, and demand generation). Taking advantage of our new system, we reviewed the monthly subnational immunization coverage in 2019–2021 in our seven priority countries. The objective was to document backslide and recovery in immunization coverage during and after the COVID-19 pandemic period. The expected outcome of this work was to contribute to regional efforts of countries’ support to identify low-performing districts early to develop strategies to improve performance before final reporting at the end of the year.

## 2. Methods

### 2.1. Data Collection

Immunization data managers at the subnational and national level of the countries’ EPI program collected immunization data on a monthly basis for all antigens. On a quarterly basis, immunization officers in respective countries’ WHO country offices shared with the immunization unit in the EMRO the monthly immunization data of all antigens based on countries’ immunization administrative coverage at the district level.

### 2.2. Data Analysis

The EMRO prepared and shared an automated spreadsheet with all WHO country offices to ensure standardization of reporting among all countries. The WHO-generated sheet had an automatic calculation of immunization coverage based on administered doses of every antigen with a subsequent color-coded level of coverage and equipped with some antigen (DTP1-DTP3 and MCV1-MCV2) dropout calculations and districts classification based on their coverage performance. The dropout rate is calculated as the proportion of drop in immunization coverage between the first and third dose of DTP or the first and second dose of MCV. WHO staff in countries filled in this spreadsheet, with an automatic calculation of immunization coverage, on a monthly basis at district and national levels. Each trimester, countries’ offices sent an updated spreadsheet. The EMRO merged data from individual countries at the end of each year, including an update of data of all quarters when applicable. As per the regular information sharing within the WHO, the EMRO shared the data regularly with the global central data repository in a database with access restricted to inside the organization. The EMRO team received data, reviewed it, and cleaned it before the analysis including an analysis for data quality issues.

The EMRO then analyzed the district-level performance as per all antigens’ coverage. Every quarter, the EMRO compared immunization coverage with the previous quarter and with the same quarter of the previous year. Zero-dose children, who are the children who never received their first dose of DTP, were analyzed separately.

At the end of every year, we analyzed the monthly coverage based on the most updated data received in the last quarter of every year. For 2019–2021, in the seven countries, we described the monthly DTP3 coverage and described the proportion of districts with at least 80% DTP3 coverage every quarter. Within every year, we compared quarters in terms of coverage. Across the years, we compared the same quarters in terms of coverage.

To estimate the coverage net gain between 2019 and 2021, we broke down the comparison by biennium. First, we calculated the difference in coverage in 2019–2020. Second, we calculated the difference in 2020–2021. Third, we plotted the net difference of the total period.

We mapped the distribution of zero-dose children at district level in the three countries with the highest number of zero-dose children for 2019–2021. Throughout the review period, for the three highest-priority countries with the highest burden of zero-dose children, we plotted by quarter the total number of zero-dose children according to DTP1 coverage.

### 2.3. Feedback

The EMRO provided feedback to the WHO country office in the form of a detailed analytic report on a quarterly basis that included sections on immunization data quality and immunization coverage analysis of all antigens. The report included conclusions of the findings and suggested recommendations for the countries’ immunization program. The EMRO then suggested that the WHO country office share the report with the immunization program counterpart for their consideration and corrective action whenever needed. The detailed analysis of the immunization coverage reflected the most important components of countries’ immunization program performance.

## 3. Results

### 3.1. Variations in Coverage throughout the Year

**Figure 1:**
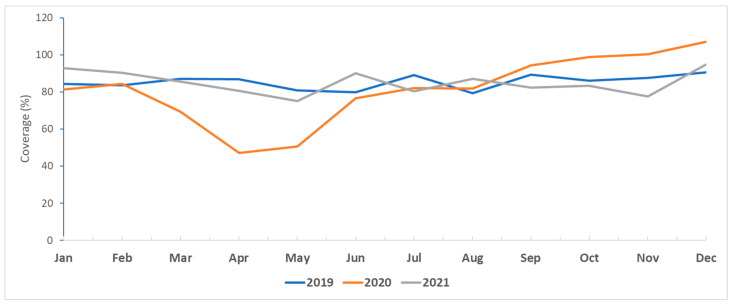
DTP3 monthly coverage in high-priority countries in the Eastern Mediterranean Region, 2019–2021. Afghanistan, Iraq, Jordan, Pakistan, Somalia, Syria, and Yemen.

**Figure 2:**
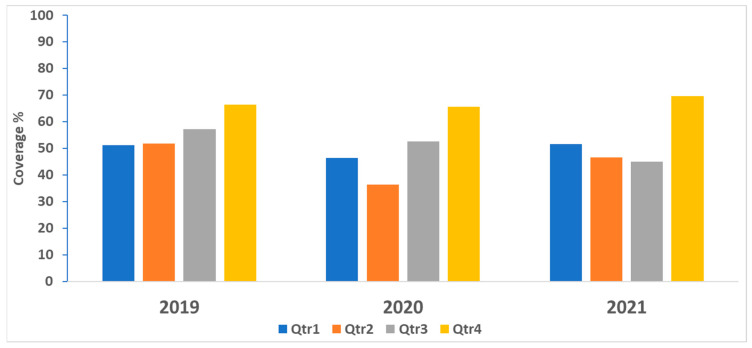
Proportion of districts with at least 80% DTP3 coverage by quarter in high-priority countries, in the Eastern Mediterranean Region, 2019–2021.

In 2020, the first year of the pandemic, the monthly DTP3 coverage dropped between March and June (Figure 1). Continuous monthly variation in DTP3 coverage occurred in the review period before the increase in the initial coverage in the last two months of 2021. The proportion of districts that exceeded 80% DTP3 coverage always improved in the last quarter every year (Figure 2). In 2021, the proportion of districts exceeding 80% DTP3 coverage was higher than in 2020. However, it did not reach the pre-pandemic performance level of 2019.

### 3.2. Recovery of Coverage following the Effect of the Pandemic

**Figure 3:**
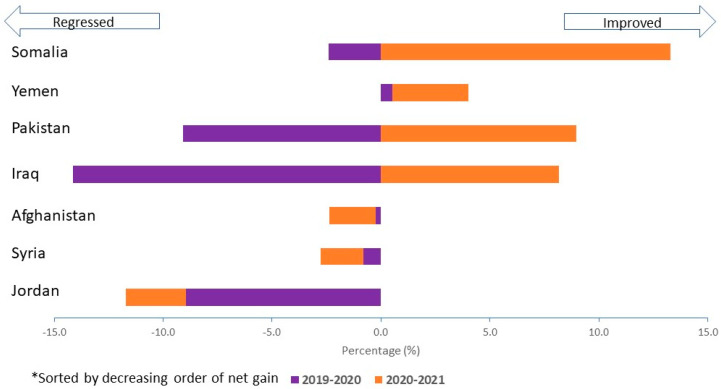
Difference in DTP3 coverage based on subnational coverage in high-priority countries in the Eastern Mediterranean Region, 2019–2021.

When we compared 2019 with 2020 and 2020 with 2021 according the DTP3 coverage net gain and net loss, three types of DTP3 coverage pattern emerged (Figure 3). First, some countries improved overall. Somalia and Yemen had a net gain of DTP3 coverage at the end of the review period because the improvement in 2021 exceeded the regression in 2020. Second, some countries recuperated just as much as they lost. DTP3 improvement in 2021 was equal to the 2020 regression in Pakistan and Iraq. Third, some countries did not recover enough. In Afghanistan, Syria and Jordan, the regression in DTP3 coverage continued in 2020 and 2021.

### 3.3. Distribution of Zero_Dose Children

**Figure 4:**
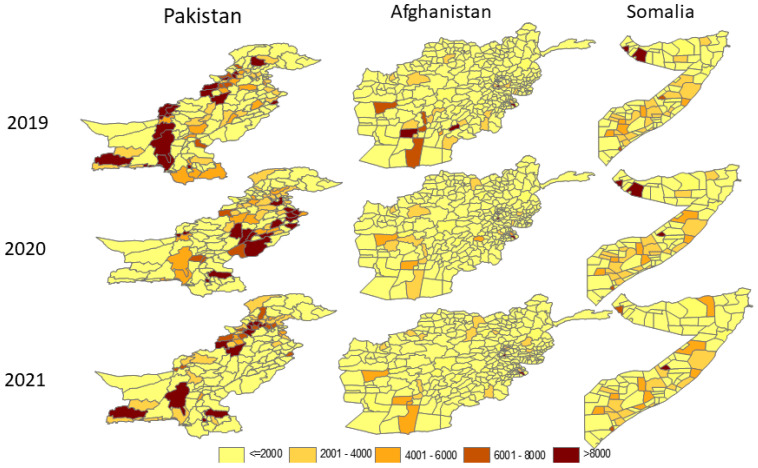
Subnational distribution of zero-dose children in the three highest burden zero-dose countries in the Eastern Mediterranean Region, 2019–2021.

The distribution of zero-dose children in the three countries with the highest burden in the region evolved over time (Figure 4). The number of districts with at least 6000 zero-dose children improved moderately in Afghanistan and substantially in Somalia throughout the follow-up period. In Pakistan, the geographical distribution differed between 2020 and 2021.

### 3.4. DTP1 Coverage

**Figure 5:**
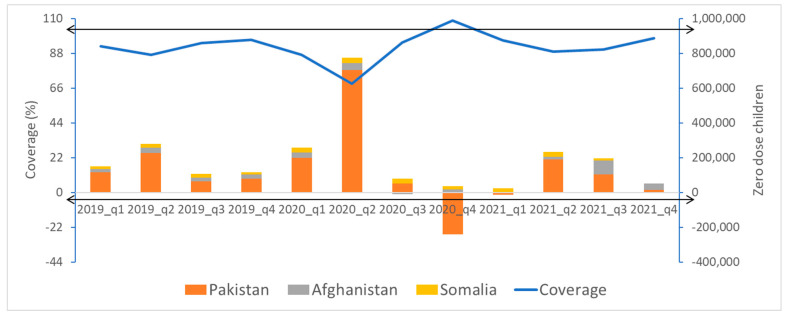
DTP1 coverage vs. zero-dose^2^ children in three high-priority countries in the Eastern Mediterranean Region, 2019–2021. ^2^Zero_dose children: DTP1-unvaccinated children. Source: monthly subnational immunization coverage data.

DTP1 coverage of the three countries with highest number of zero-dose children reached 109% in Q4 of 2020 after a sharp drop to 69% in Q2 of 2020. However, the number of zero-dose children in Pakistan decreased to 1/10 of its burden in 2021 at Q4. In Afghanistan, the number of zero-dose children more than doubled throughout 2021 (Figure 5).

## 4. Discussion

There was a sharp drop in DTP3 coverage from March to June in 2020, the first year of the pandemic when countries started to sift attention to the pandemic control measures, and most of the immunization staff were concentrating on working on this initial phase of the outbreak response. Afterwards, it started to recuperate by the end of the year. The pre-pandemic level of DTP3 coverage continued to improve throughout the review period. Another study analyzing the disruption and recovery of immunization services in 170 countries depicted a similar trend [10]. More generally, in 2019–2021, the low proportion of districts achieving at least 80% DTP3 coverage at the beginning of the year started to improve in the second half of the year, with the best performances during the last quarter of every year. Countries identified missed children throughout the first three-quarters to guide the catching up of under-immunized children during the fourth quarter of every year. Some high-priority countries in the region reported coverage exceeding 100%. Experience from other countries suggests that this is mainly due to the inability to estimate the correct denominator at the district level. Other reasons could include the catchup of a backlog of unimmunized children in one month or quarter of the year [11,12]. In 2021, the performance of districts followed the same improving trend in terms of the proportion of districts reaching 80% DTP3 coverage by the end of the year. However, in the high-priority countries, coverage did not reach the pre-pandemic level of 2019. This delay varied in magnitude between countries involved in the analysis. Countries’ programs to improve immunization coverage also competed with the introduction of COVID-19 vaccination and were taking place under stretched conditions of the ministries of health generally and the immunization programs specifically, sometimes aggravated by humanitarian situations in some countries, which increasingly needed more effort to cope with those situations [13].

Immunization service delivery adapted to the pandemic differently from country to country. These differences depended on the agility of the health system and the performance of one or more of the different components of expanded programs on immunization (i.e., governance, work force, finance, vaccine and cold chain, immunization service delivery, surveillance, and demand generation). The EMRO initiative to analyze monthly subnational immunization coverage on a quarterly basis provided support to the stretched country system. It facilitated the identification of unimmunized and under-immunized children before the end of the year to prevent a larger backsliding of immunization coverage. Backsliding of immunization, due to the pandemic, has been depicted in almost all countries in the world regardless of their income. Low- and middle-income countries have been most affected in terms of vaccination coverage of most of the antigens [14]. The World Health Organization [15] provided guidance on aspects of immunization recovery and catchup of missed children. This called for strengthening routine service delivery, conducting antigen specific or multiantigen campaigns and undertaking Periodic Intensification of Routine Immunization (PIRI). Somalia and Yemen responded early to the reduction in DTP3 coverage in 2020. The 2021 coverage reflected this response. In Pakistan and Iraq, the regression in DTP3 coverage in 2020 was compensated in 2021. Instant corrective measures were taken to address the backslide in immunization in high-priority districts based on their identification in the subnational coverage analysis. Afghanistan, Syria, and Jordan continued to regress throughout the period until the last quarter of 2021. This was mainly attributed to the delayed response of immunization service recovery in the lowest-performing districts.

Almost all EMR high-priority countries depend on outreach and mobile approaches that support fixed centers to deliver immunization services. The cancellation of outreach services following the COVID-19 lockdown meant that many children missed vaccination which resulted in backsliding of the immunization coverage and magnification of the number of zero-dose children [16]. Gavi in its support for low- and middle-income countries, among which included countries in this analysis, emphasized reaching zero-dose children at the subnational level (i.e., the last administrative level where data could be generated and reported). However, reaching zero-dose communities within districts becomes a long term goal, when identifying the target of zero-dose children in those communities becomes available [17]. Our analysis has confirmed that immunization coverage at the national level masks the heterogeneity of the coverage at the district level on a monthly basis in the reported countries. Hard-to-reach areas are the most affected areas with poor immunization coverage and host the biggest number of zero-dose children in all countries [18].

The constant decrease in the number of zero-dose children in the same districts throughout the years has led to an overall decrease in the number of zero-dose children in countries. This improvement is mainly related to the timely implementation of catch-up multi-antigen campaigns or an enhanced immunization outreached approach at the subnational level. These campaign and enhanced outreached strategies succeeded in catching up with underimmunization so more children have been reached with all missing antigens within a reduced timeline. Changes in geographical location in zero-dose children in Pakistan is related to different responses by districts and to substantial mobile populations between districts and provinces. Similar findings were also depicted in other similar-income countries [16,19]. In other settings [20], missed children are constantly located in the same districts and provinces due to a continuous lack of resources allocated and available to the expanded program in immunization. In Pakistan, the same number of districts had the same number of zero-dose children in 2019–2020, but the location changed. Yet, overall districts with zero-dose children have much decreased throughout the last quarter of 2021 due to a decrease in the overall number of zero-dose districts nationwide. In Afghanistan, the situation was very different. The number of zero-dose children increased in 2021, despite the decrease in zero-dose children in a number of districts compared to the great increase in zero-dose children in a different number of districts. This is mainly related to the worsening of the immunization performance and increased number of zero-dose children in several other districts. In the Laghman province of Afghanistan, Abid et al. reported [21] a period of improvement in immunization performance in most of the 400 districts through 2020 and 2021 during the pandemic period. An earlier study [22] did not suggest that the conflict situation in Afghanistan significantly affected DTP3 immunization coverage in most Afghanistan districts. However, the conflicts referred to were shorter than the pandemic. Finally, Somalia improved throughout the last two years of the review period, which resulted in recuperation of unimmunized and under-immunized children vaccination because of their early response since the first year of the pandemic. Focusing on reaching zero-dose children is the ultimate objective for reaching the sustainable development goals [23].

Our review suffered from several limitations. First, countries lacked accurate estimations of their monthly immunization target. This led to coverage exceeding 100% for several antigens in some districts, with a subsequent impact on the overall estimate of national immunization coverage. This has led to reflecting a negative number of zero-dose children in Q4 2020. The WHO [24] formulated guidelines for the estimation of denominators to accurately calculate the immunization coverage. The different methods to calculate denominators can be customized to the country context. Second, inaccurate and delayed reporting of monthly immunization data along with the existence of mobile, migrant and internal displaced populations (IDPs) may reflect unrealistic recovery of immunization coverage in some districts. Because of the inaccurate estimation of the target population, frequent mapping of mobile populations to include them in the denominator of the districts’ catchment area will improve the estimation of immunization-targeted children. Third, cross-district vaccination and geographical redistribution of some districts may have impeded the real estimation of the subnational burden of zero-dose children, especially in countries with no cross notification of immunization coverage on a monthly basis. This hindered effective planning of catchup immunization campaigns. Electronic immunization registries with instant registration of immunization, which could be monitored centrally, and GPS-based home-based records could help in identifying missed children in a timely manner.

## 5. Conclusions

Based on this review, we can draw several conclusions. First, DTP3 coverage decreased in 2020, in the first year of the COVID pandemic, and recovered in the fourth quarter of every year. Second, countries differed in their recovery in 2019–2021, with a positive net improvement in DTP 3 coverage, a compensation, or a sustained reduction. Third, the geographical distribution of districts with a high number of zero-dose children is dynamic from year to year. While some hotspots are being addressed, new ones emerge. Based on these conclusions, we recommended the following actions. First, we need to monitor administrative monthly immunization coverage data of all antigens with evaluation of the dropout rates at the subnational level to detect low-performing districts and plan for catchup. Second, we need to identify bottlenecks towards reaching unvaccinated children at the subnational level based on the number of unimmunized and under-immunized children with a high focus on the zero-dose children. Third, we need to customize strategies continuously to improve the coverage of the districts with a high number of zero-dose children throughout the four quarters every year and monitor the progress of other better-performing districts. In countries with highly populated districts, there is a need to address subdistrict level data so zero-dose children can be located at the community level. This analysis was proposed by the EMRO as a model approach. Ultimately, the management of this approach should be transferred to countries so that the national immunization programs own the process and start to have a close monitoring of the immunization coverage on a monthly basis for up-to-date consideration should districts show a decline in the immunization coverage.

## Data Availability

Data is available at WHO country office of contributing countries and at Eastern Mediterranean Regional office of WHO.
